# Neuromuscular Monitoring and Wake-Up Test During Scoliosis Surgery

**DOI:** 10.7759/cureus.44046

**Published:** 2023-08-24

**Authors:** Saely Paunikar, Amreesh Paul, Dnyanshree Wanjari, Nitin R Alaspurkar

**Affiliations:** 1 Anaesthesiology, Jawaharlal Nehru Medical College, Datta Meghe Institute of Higher Education and Research, Wardha, IND

**Keywords:** direct cortical stimulation-motor evoked potential (dcs-mep), somatosensory evoked potentials (ssep), wake-up test, scoliosis surgery complications, scoliosis surgery, adult idiopathic scoliosis, neuromuscular monitoring

## Abstract

A typical spine is straight and symmetrical, with all of the vertebrae facing forward when viewed from the posterior. Scoliosis is a term used to describe a lateral deviation of the spine's normal vertical line that is greater than 10° on an X-ray. More severe curves are sometimes accompanied by medical issues such as increased back discomfort and cardiorespiratory difficulties along with the cosmetic components of the deformity. The test for spinal cord integrity prior to the widespread adoption of intraoperative neurophysiologic monitoring (IOM) was the wake-up test. In this article, we review the challenges faced by anesthesiologists and surgeons during intraoperative monitoring and the importance of clinical assessment of surgical outcomes.

## Introduction

Scoliosis is a complex spinal malformation that affects the lateral curve of the spine in the sagittal, axial, and coronal planes and is a complex, three-dimensional rotational malformation [1]. It could be idiopathic (most common form), neuromuscular (cerebral palsy, spinal muscular atrophy, spina bifida, etc.), or congenital [2]. It is seen in 2-3% of the total population, and it begins its manifestation early in childhood or early adolescence and becomes more prevalent as the years pass by [3]. It is more often seen in females than males; in females, it requires surgical correction more often than in males. In 90% of cases, the patients present with back pain as the primary complaint, and the initial treatment is conservative with physiotherapy or braces. The deformity of the spinal cord can become severe, limiting routine activities and necessitating surgery to correct the deformity [4]. Advanced scoliosis may be associated with significant pulmonary and cardiovascular dysfunction. A thorough preoperative examination must be done to assess existing neurological status, exercise tolerance, and pulmonary function test, which may reveal restrictive lung diseases [5]. Scoliosis correction surgery involves realigning the vertebrae with metal rods, screws, and hooks. One major complication associated with scoliosis surgery is injury to the spinal cord and following neurological deficit [6]. The Stagnara wake-up test is used intraoperatively to assess the spinal cord's functional integrity. Apart from the wake-up test, various techniques of neuromuscular monitoring are available, which include somatosensory evoked potentials (SSEPs), motor evoked potentials (MEPs), and electromyography (EMG). Luk has recommended using sensory evoked potentials and MEPs for scoliosis surgery [7]. In this case report, we present the case of a 23-year-old female with restrictive lung disease posted for scoliosis surgery.

## Case presentation

**Figure 1:**
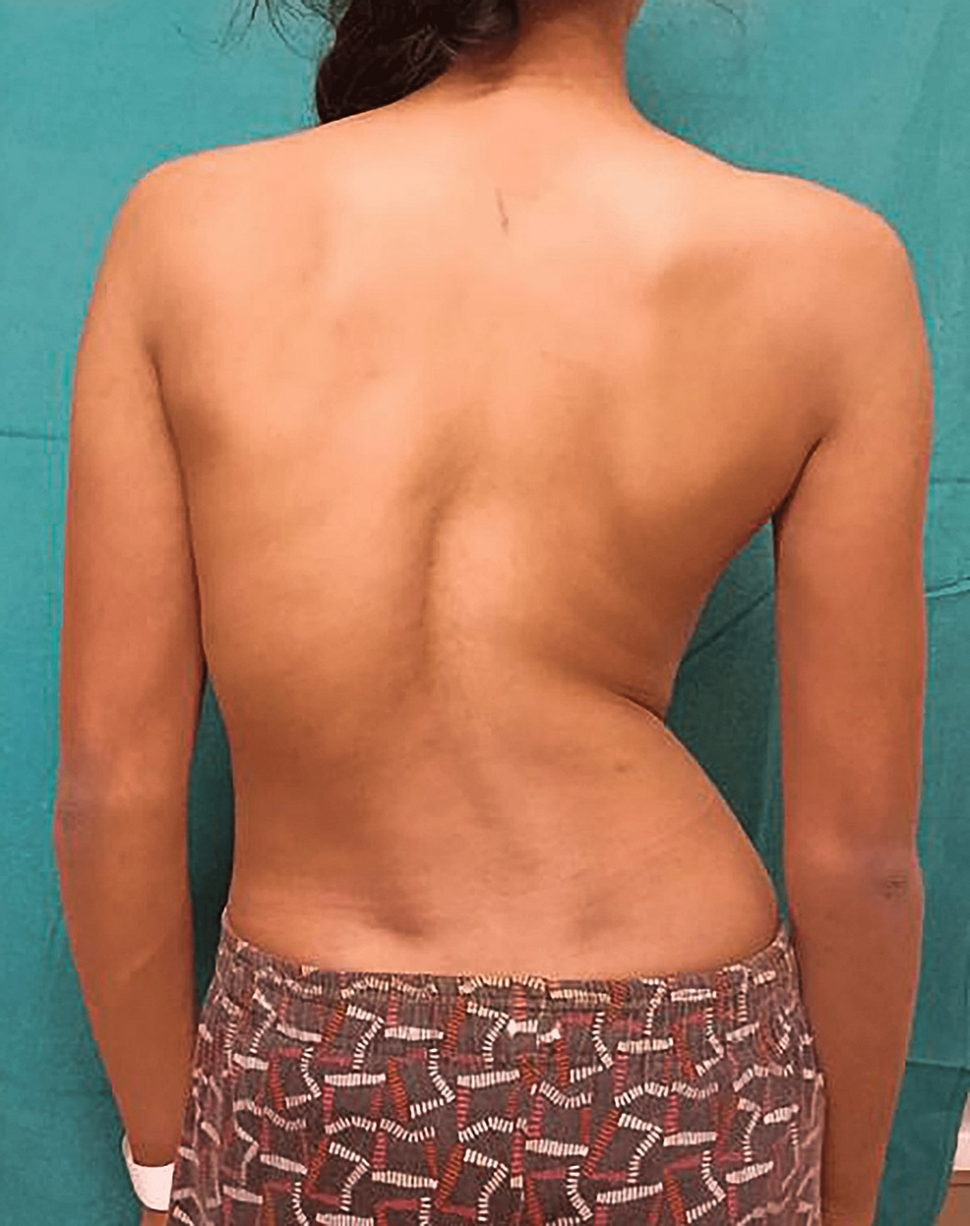
Clinical image of the patient showing altered lateral curvature of the spine.

A 23-year-old female presented to our hospital with a history of breathlessness during strenuous activity for three months. The patient gave a history of idiopathic scoliosis since early childhood (Figure 1). The deformity gradually progressed over the years and was associated with lower back pain, difficulty walking, and weakness of the lower limbs. She developed breathlessness during strenuous activities for the last three months due to worsening her scoliosis.

**Figure 2:**
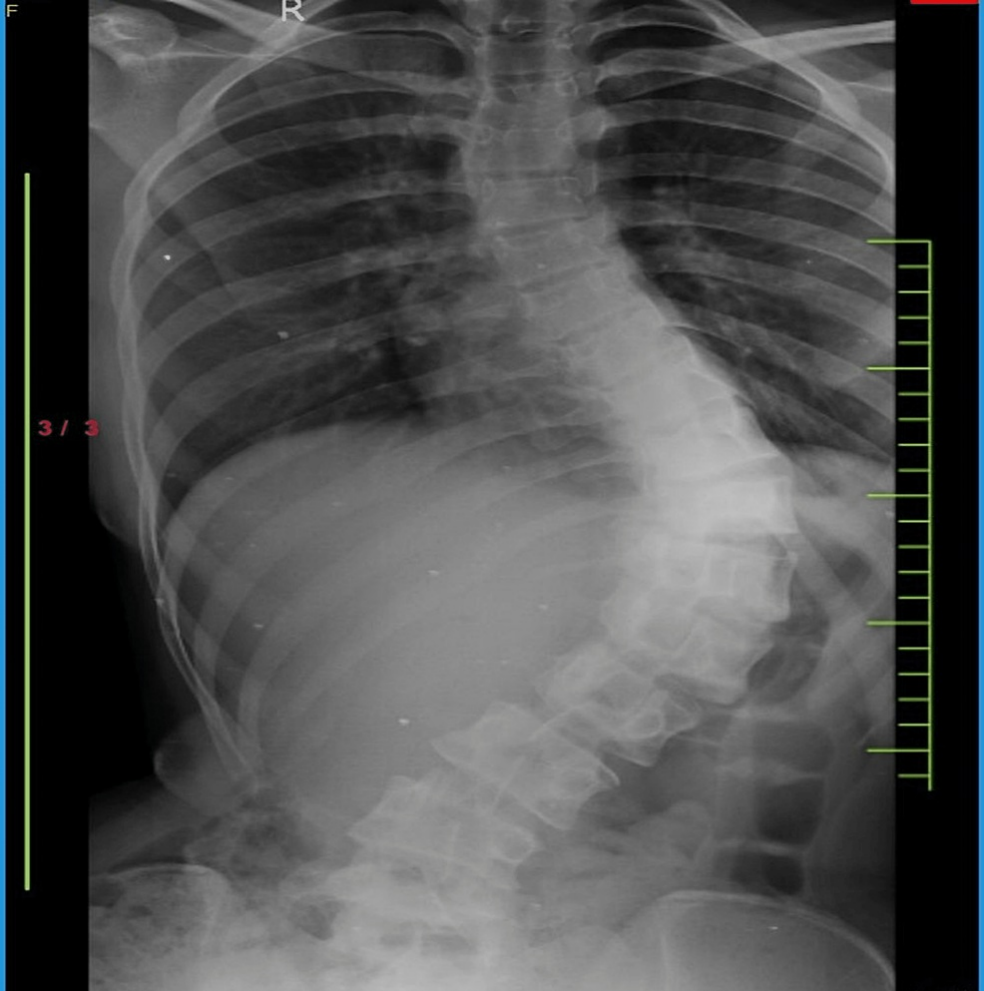
Preoperative X-ray showing the degree of scoliosis.

Given the worsening symptoms, she was planned for scoliosis correction surgery, and a pre-anesthetic evaluation was conducted. The patient exhibited New York Heart Association (NYHA) class II functional capacity. She had a pulse rate of 84 bpm, respiratory rate of 21/min, blood pressure of 110/80 mmHg, and a room air saturation of 94%. Airway examination revealed an inter-incisor distance of 3 cm, Mallampati class II, and restricted neck movements. Auscultation of the respiratory system revealed clear breath sounds in all lung fields. The pulmonary function test revealed a restrictive pattern (forced expiratory volume in one second (FEV1)/forced vital capacity (FVC) >75% of predicted value, FVC 56% of predicted value). Blood investigations were within normal limits. An X-ray of the spine was taken (Figure 2). The patient was informed about the administration of general anesthesia for the surgery, along with the associated benefits, risks, and complications. She was also briefed about the neurological deficit that can arise as a complication of the surgery. She was informed about the plan for neurological monitoring and the implementation of the wake-up test if required, and informed and written consent for the same was obtained.

**Figure 3:**
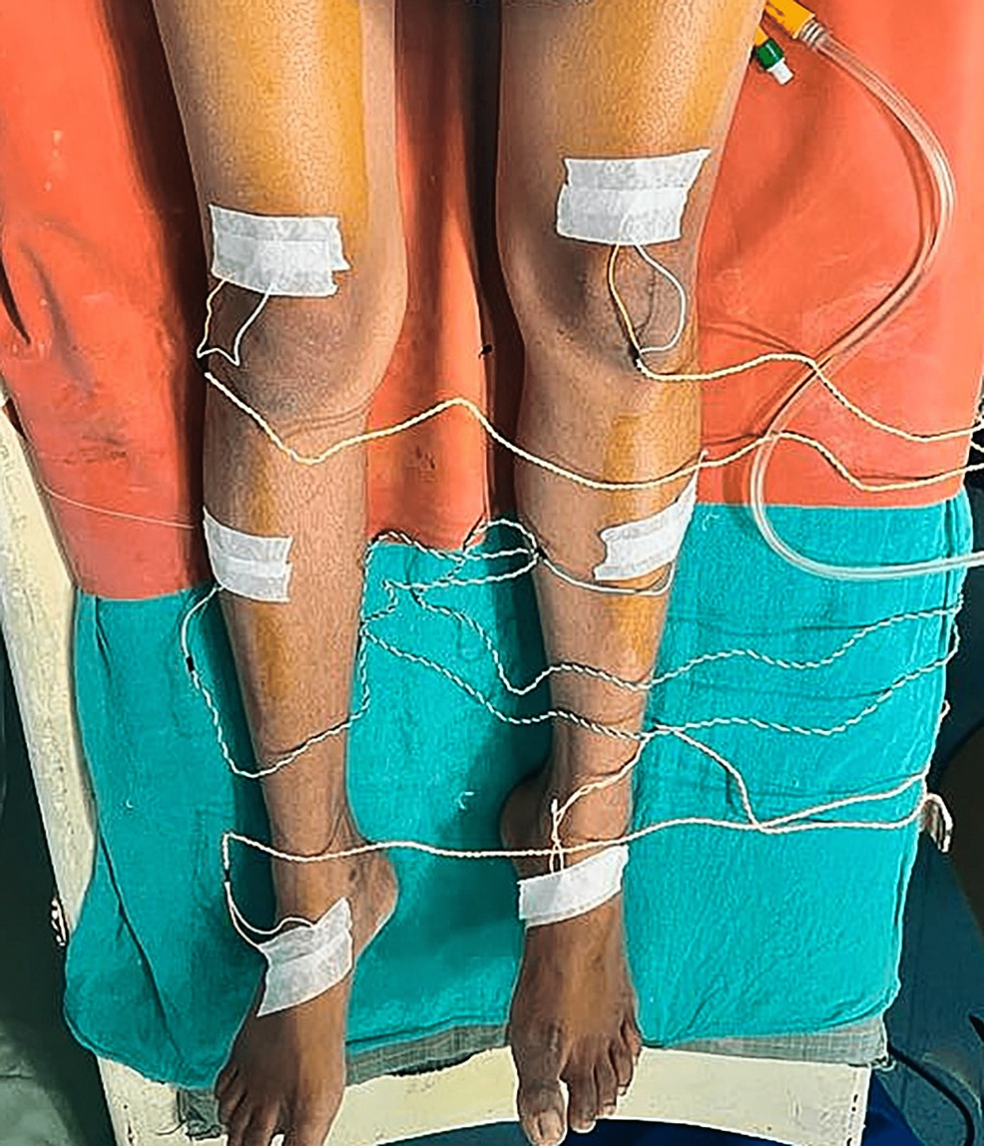
Electrode placement.

**Figure 4:**
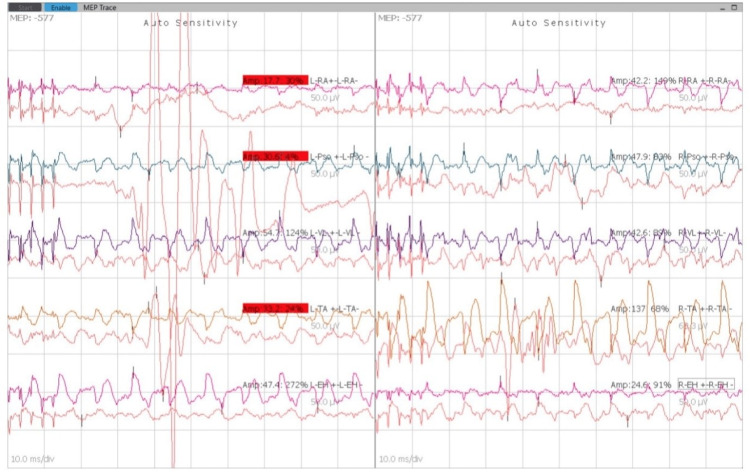
Baseline MEP readings. MEP: motor evoked potential.

The patient was kept nil per oral per standard guidelines the day before the surgery. The patient was evaluated in the preoperative room and shifted to the operation theater. Non-invasive monitors were attached, vital baseline parameters were noted, and a 20-gauge intravenous line was secured in the left upper limb. The patient was premedicated with glycopyrrolate 0.2 mg, midazolam 2 mg, and fentanyl 100 mcg intravenously. The patient was administered intravenous propofol 100 mg and succinylcholine 100 mg and intubated with a cuffed endotracheal tube with an internal diameter of 7.5 mm. The position of the tube was confirmed by capnography and five-point auscultation. A 7-Fr central venous catheter was inserted into the right jugular vein using the Seldinger technique. Neurotechnicians attached electrodes for monitoring SSEP and MEP (Figure 3), and the baseline MEP readings were recorded (Figure 4). Leads were connected bilaterally into the rectus abdominis, psoas major, vastus lateralis, tibialis anterior, and extensor hallucis longus. Two leads were inserted into the scalp over the parietal bone, and one earthing lead was inserted into the gluteal region.

**Figure 5:**
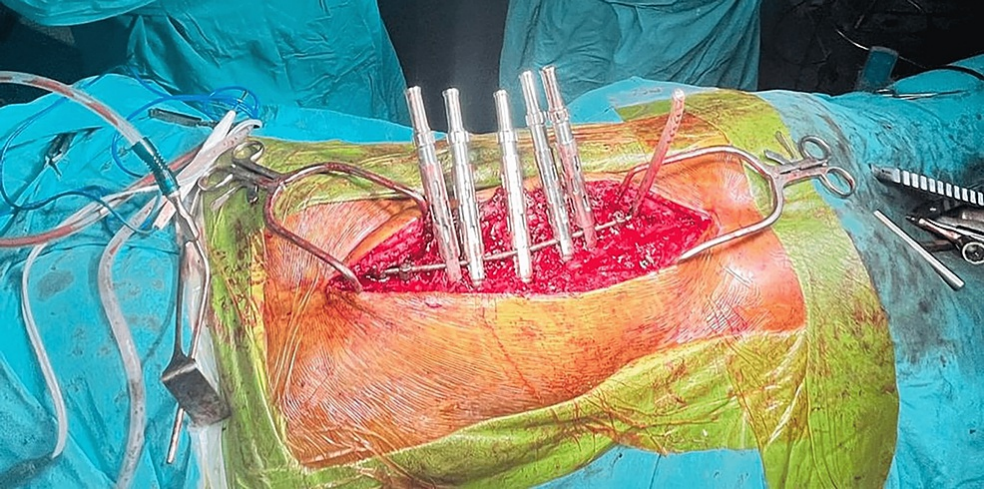
Instrumentation.

**Figure 6:**
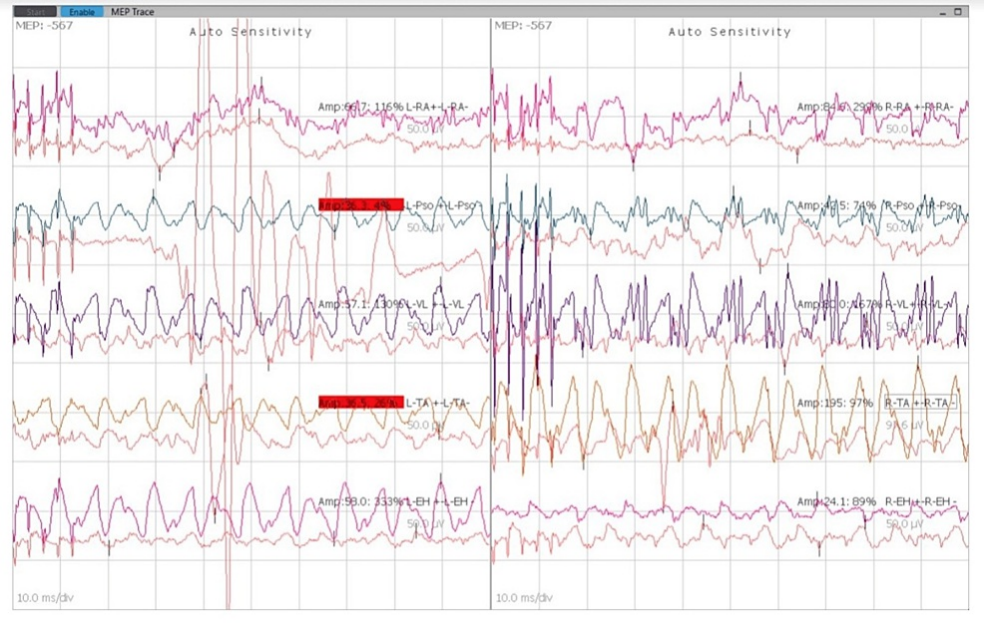
MEP readings during the process of instrumentation at the lumbar level. MEP: motor evoked potential.

**Figure 7:**
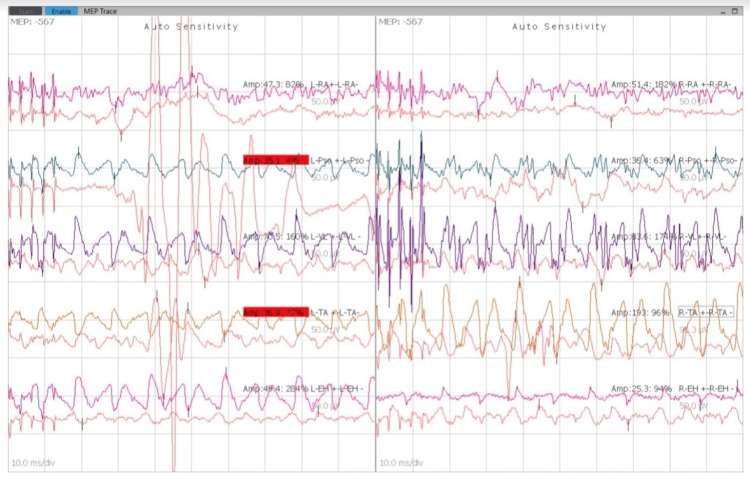
MEP readings during the process of instrumentation at the T12 level. MEP: motor evoked potential.

Vecuronium 6 mg was given intravenously, and the patient was maintained on oxygen, nitrous oxide with a FiO_2_ of 50%, and sevoflurane. The patient was positioned prone for the procedure. Adequate padding of the eyes and care of pressure points were taken. An intravenous propofol infusion was started at 100 mcg/kg/min. The mean arterial pressure was kept above 70 mmHg to provide adequate perfusion to the spinal cord. The minimum alveolar concentration was maintained at 0.6 throughout the surgery to prevent interference with the neuromuscular monitoring. Under strict aseptic precautions, the parts were painted and draped. Approximately a 30 cm incision was taken extending from the lower border of T1 up to S1. Layers were dissected, and the vertebrae were reached. Pedicle screws were inserted starting from the bilateral L4 level, followed by L3 and L2 levels. SSEPs and MEPs were checked post-lumbar fixation and found to be the same as the baseline. Following this, bilateral T12 and T10 levels were fixed with pedicle screws, and electrical activity was rechecked, which was found to be normal. Subsequently, the right T7, bilateral T6, and T5 levels were fixed with pedicle screws. SSEPs and MEPs were checked and found to be the same as the baseline. A long connecting rod was put through the pedicle screws on the right side (Figure 5), and the deformity was corrected. SSEPs and MEPs were checked after the deformity correction and found to be the same as the baseline (Figures 6, 7). Analgesia was maintained with intravenous fentanyl 50 mcg/h throughout the surgery. A total blood loss of approximately 1100 mL was replaced with two units of packed RBCs.

**Figure 8:**
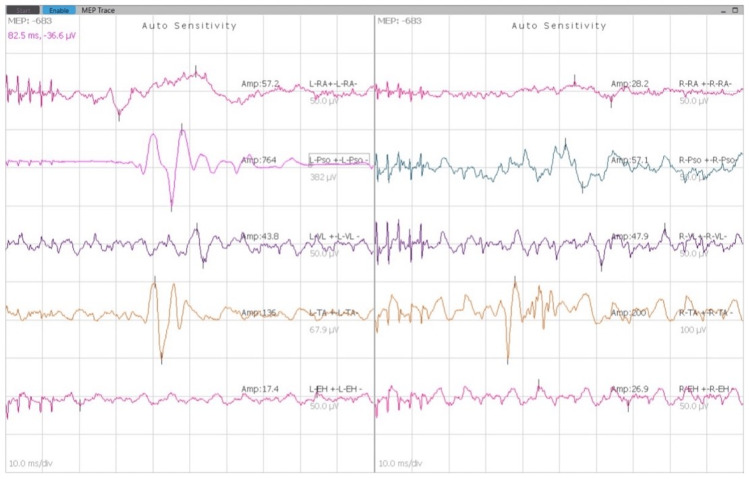
MEP recordings showing inadequate response. MEP: motor evoked potential.

**Figure 9:**
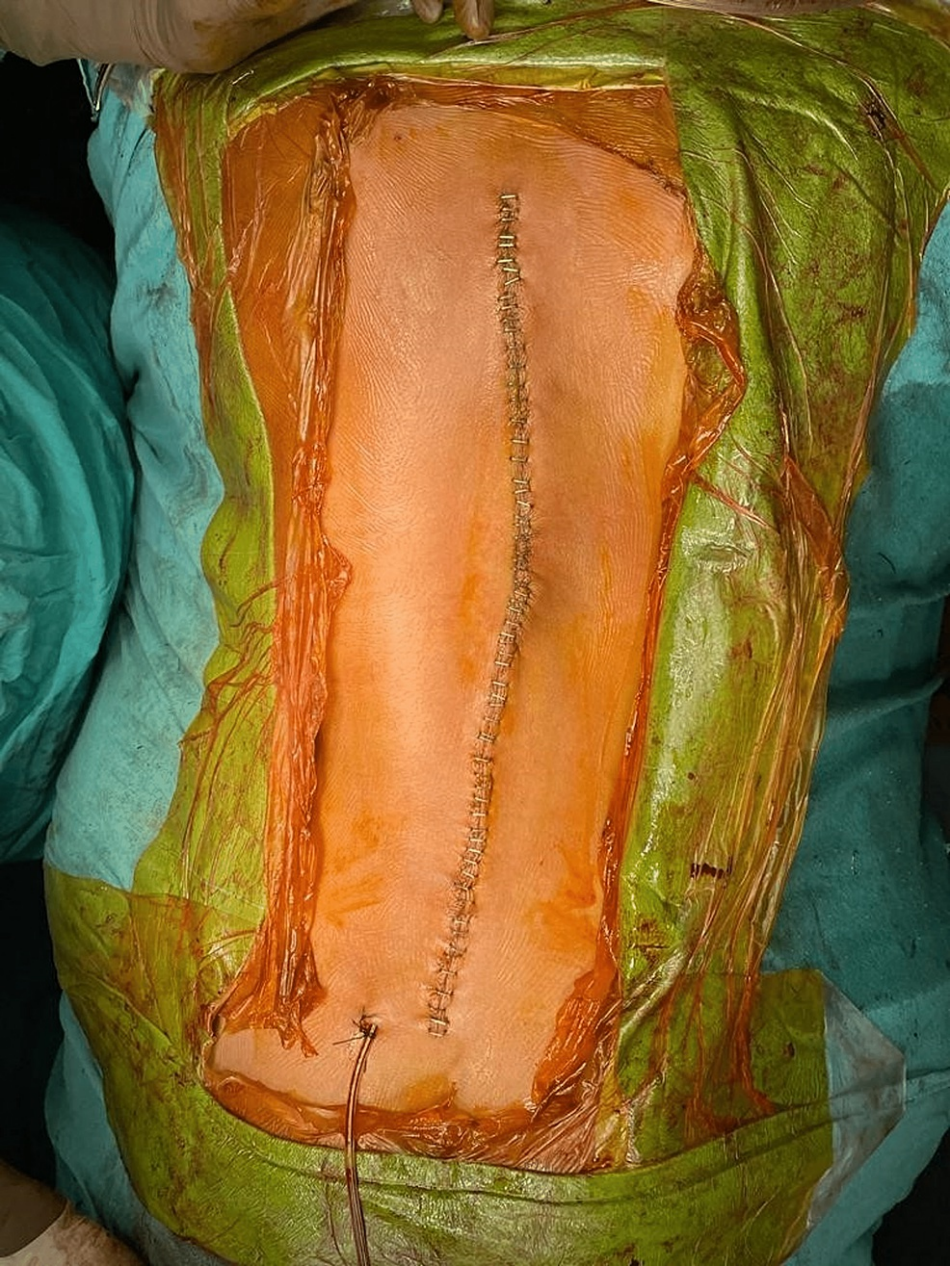
Image taken after the completion of the surgery.

Another connecting rod was inserted into the left side, and the correction was done. Final MEPs were repeated and did not record an adequate response (Figure 8). All anesthetic agents were cut down to check if they were causing interference with the monitoring. Since this did not yield a satisfactory response in the neuromuscular monitoring, it was decided to do a wake-up test to check for any neurological damage. Under adequate analgesic cover of intravenous fentanyl, the patient was woken up and asked to wiggle the fingers of her upper and lower limbs. After a positive outcome of the wake-up test was obtained, she was given vecuronium intravenously, and the surgery was continued. Fixation was checked under fluoroscopy and found to be satisfactory. A thorough wash was given, and a drain was inserted. The closure was done in layers using Vicryl 0 and a skin stapler (Figure 9). Sterile dressing was done.

**Figure 10:**
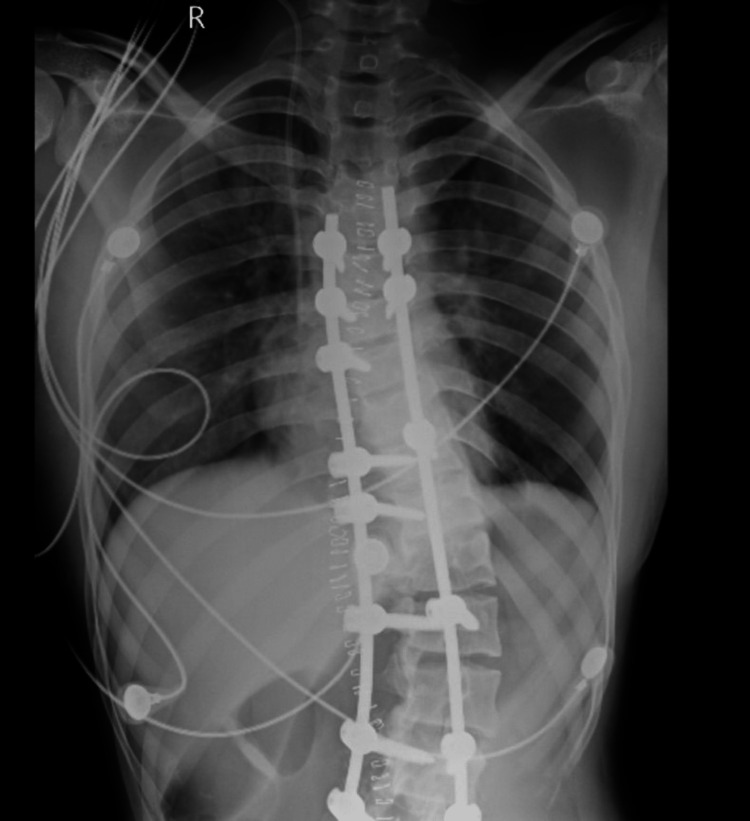
Postoperative chest X-ray showing a central venous catheter in the right jugular vein.

**Figure 11:**
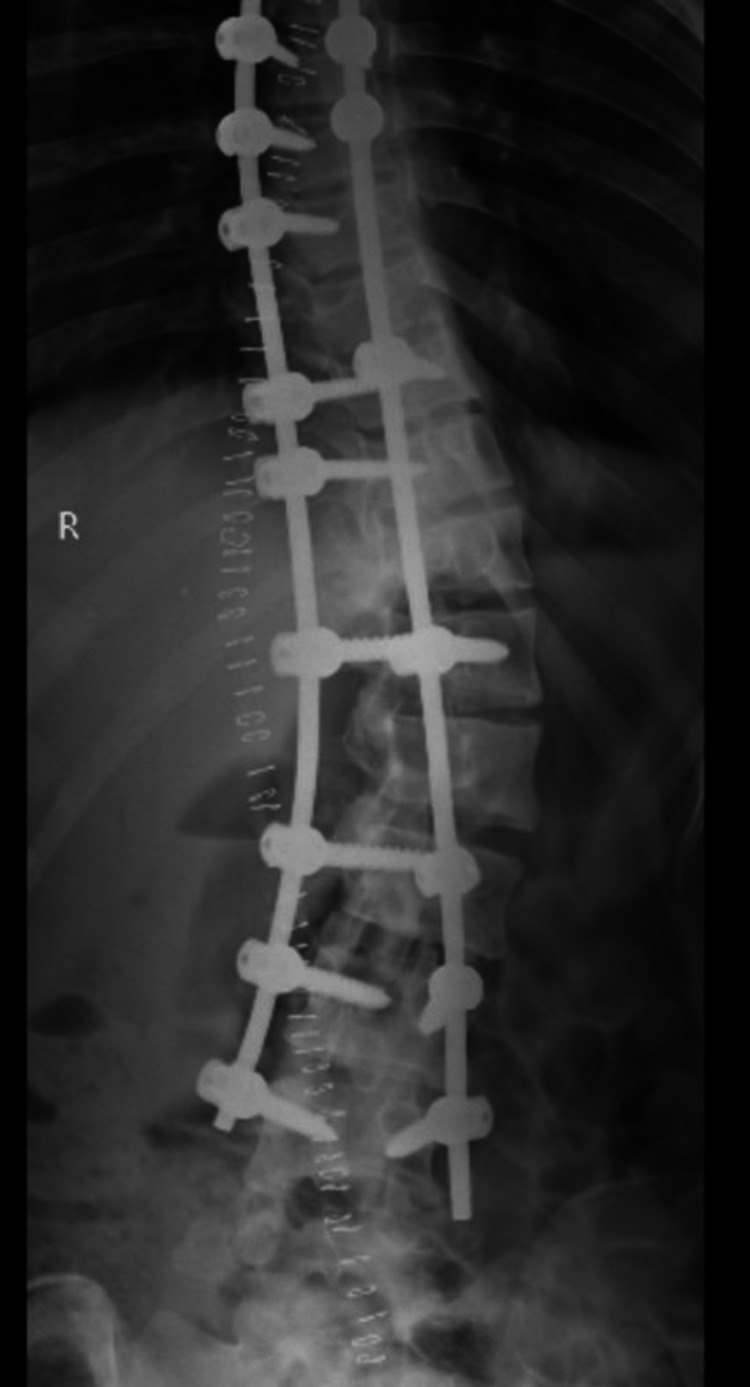
Postoperative imaging showing rods and screws after surgery.

An arterial blood gas (ABG) was done to rule out any metabolic disturbances and showed normal results. After the surgery, the patient was made supine and extubated. The patient was shifted to the post-anesthesia care unit, where she was monitored for 12 hours before being shifted to the ward. Postoperative blood investigations and chest radiographs were normal (Figures 10, 11).

The patient was evaluated for pain, analgesic needs, and any memories of the procedure before being discharged five days after surgery following an uncomplicated recovery.

## Discussion

Scoliosis is a complex spine deformity resulting in lateral curvature, rotation of the vertebrae, and a deformity of the rib cage. The thoracic or lumbar regions of the spine frequently curve to one side or the other. Less than one in 1,000 children aged 6-14 years has clinically significant scoliosis, despite the condition being seen as a prevalent issue in children. The degree of spinal curvature and the angle of trunk rotation both affect how severe scoliosis is [3]. The respiratory, cardiovascular, and nervous systems are typically secondarily affected. This highlights the significance of prompt orthopedic care for patients in this group [8]. The measurement of Cobb's angle is the most accepted method for measuring spinal abnormalities and curvature from a spine X-ray in a case of scoliosis. The Cobb's angle is formed by lines drawn at the upper and lower borders of the most twisted vertebrae above and below the curve's apex, respectively [9].

For patients with scoliosis, it is critical to prepare for any potentially difficult airway management scenarios. Bleeding is another major issue that affects people who have scoliosis surgery. After posterior correction of scoliosis, the intraoperative wake-up test is a classic method for detecting neurologic issues [10]. It was first described in 1973 by Vauzelle et al. The wake-up test, commonly known as Stagnara test, is a simple method to identify lower limb voluntary motor function. It is a crude examination of spinal motor capacity. It continues to be the most accurate evaluation of an intact spine for several reasons [11]. The intraoperative awakening test can be used alone or in combination with electrophysiological monitoring, being a beneficial adjunct when the latter shows deteriorating responses or to confirm positive electrophysiological data.

MEPs are the electrical activity detected in peripheral nerves and muscles following cortical or spinal stimulation. MEPs seem to offer a helpful indicator of the functional integrity of motor pathways. SSEP signals may be suppressed by anesthetics or impossible to obtain in some patient situations, including neuromuscular degeneration, and missed by SSEP monitoring in the case of an anterior cord injury. The SSEP test keeps track of the neural networks that regulate pressure, touch, temperature, and pain perception. SSEP monitoring evaluates the signal that is transmitted to the brain and provides neuromonitoring real-time feedback [12]. The patient should be informed of the wake-up test during the pre-anesthesia visit and it should be planned well in advance. One hour prior to the anticipated wake-up time, if volatile anesthetics are being administered, they should be discontinued. Despite advanced neuromuscular monitoring being available now, clinical assessment yields a much better surgical outcome and decreased neurological complications.

## Conclusions

Patients planned for the surgical correction of scoliosis pose a wide variety of challenges for anesthesiologists and surgeons. A holistic approach must be employed to manage these patients to provide the best possible patient care with the least possible complications. With recent advances in neuromuscular monitoring, the neurological complications associated with the surgery can be decreased. Clinical assessment in the form of the wake-up test under adequate analgesic cover may be needed despite the fact that such advanced monitoring is available. In this case, neuromuscular monitoring may provide less than adequate responses. Hence, a combination of monitoring techniques and clinical tests may be required to obtain the best possible results. Thus, we would like to conclude that using the wake-up test to check for the integrity of the spinal cord remains a useful test to resolve uncertainties in intraoperative neurological monitoring or when these monitoring techniques are unavailable.
